# Adverse obstetric and neonatal outcomes of adolescent pregnancies in Africa: a scoping review

**DOI:** 10.1186/s12884-022-04821-w

**Published:** 2022-07-27

**Authors:** Mustapha Amoadu, Doris Hagan, Edward W. Ansah

**Affiliations:** Department of Health, Physical Education and Recreation, University of Cape Coast, Cape Coast, Ghana

**Keywords:** Adolescent pregnancy, Adverse pregnancy outcomes, Risk factors, Africa

## Abstract

**Background:**

Adolescent pregnancy is a public health issue with well-defined causes and health risks with social and economic implications. Aim of this review was to examine adverse pregnancy outcomes and risk factors associated with adolescent pregnancy in Africa.

**Method:**

PubMed Central, Science Direct and JSTOR were the main databases for the literature review. Other online sources and experts were consulted for relevant studies. In all, 11,574 records were identified and 122 were considered as full-text studies for evaluation after thorough screening and removal of duplicates. Finally, 53 studies were included in this review for thematic synthesis.

**Results:**

The 53 studies sampled 263,580 pregnant women, including 46,202 adolescents (< 20 years) and 217,378 adults (> 20 years). Adolescent pregnancy was associated with higher risks of adverse pregnancy outcomes. Factors of poor pregnancy outcomes included low socioeconomic and educational status, poor utilization of antenatal care, risky lifestyles such as alcohol consumption, and unattractive health care factors. Maternal health care utilization was identified as an important factor to improve pregnancy outcomes among adolescents in Africa.

**Conclusion:**

To prevent adolescent pregnancy, stakeholders need to help lower socioeconomic inequalities, poor utilization of antenatal care, alcohol consumption, and improve adolescents’ health care and their educational status. Issues such as child marriage, abortion, poor health care infrastructure and non-adolescent friendly health facilities need to be addressed.

**Supplementary Information:**

The online version contains supplementary material available at 10.1186/s12884-022-04821-w.

## Background

The stage of adolescence, ages between 10 and 19, is critical and must be a concern to every person, parents, schools and governments [1]. They account for approximately 16% (1.2 billion) of the global population [1]. In 2015 alone, approximately 1.3 million adolescents died worldwide, resulting in approximately 3561 deaths per day [2]. It is worth noting that two-thirds of these deaths occurred in low-income countries [2]. Every year, approximately 21 million adolescent girls become pregnant in low-income countries, with approximately 12 million of them giving birth [3]. Furthermore, 2.5 million adolescent girls under the age of 16 give birth each year in these countries [4]. Recent data suggest that approximately 777,000 adolescent girls under the age of 15 give birth each year at a rate of 43 births per 1000 girls globally [5]. This requires provision of quality education, health care and well-being opportunities for all citizens as well as to ensure healthy lives and well-being for all at all ages [6].

Complications of pregnancy, childbirth and abortion are the leading causes of death among adolescent girls [5]. High prevalence of hypertensive disorders, low birth weight and preterm birth in adolescent pregnancies have been reported in studies from both high and low-income countries [7]. Adolescents are more likely to have unwanted pregnancies, and those who become pregnant are typically from low-income families, are less educated and live in rural areas [1, 2, 4, 7, 8]. The relationship between adverse pregnancy outcomes and adolescent pregnancy has been a source of debate, whether adverse pregnancy outcomes in adolescents are caused by age, a risky lifestyle, lack of access to maternal health care, pre-existing health conditions or adolescents’ poor socioeconomic and demographic status [7, 9–11]. The prevalence of adverse perinatal outcomes such as low birth weight (LBW) and preterm birth also vary significantly across geographical areas [10, 12].

This scoping review included studies that looked at sub-group risk factors and adolescent pregnancy outcomes. Furthermore, due to geographical variations in pregnancy outcomes, this scoping review focused on Africa, which has the highest prevalence of teen pregnancy globally, with approximately 28% of girls in West and Central Africa and about 25% in Southern and Eastern Africa giving birth before the age of 18 [13]. Even though recent reviews on adolescent pregnancy and its related adverse obstetric and neonatal outcomes have included a few studies from Africa, they did not review evidence on risk factors that predispose adolescents to adverse pregnancy outcomes [7, 14]. This scoping review aimed at examining whether childbearing before age 20 in Africa is associated with increased adverse maternal and neonatal outcomes. The review also explored evidence regarding risk factors associated with adverse pregnancy outcomes.

## Methods

This scoping review was based on the guidelines of Arksey and O’Malley [15]. We also applied the preferred reporting items for systematic reviews and meta-analyses extension for scoping reviews (PRISMA-ScR) checklist [16]. The research questions included: (1) what are the adverse obstetric and perinatal/neonatal outcomes associated with adolescent childbirth in Africa? and (2) what are the risk factors associated with adverse pregnancy outcomes among adolescents in Africa?

**Fig. 1:**
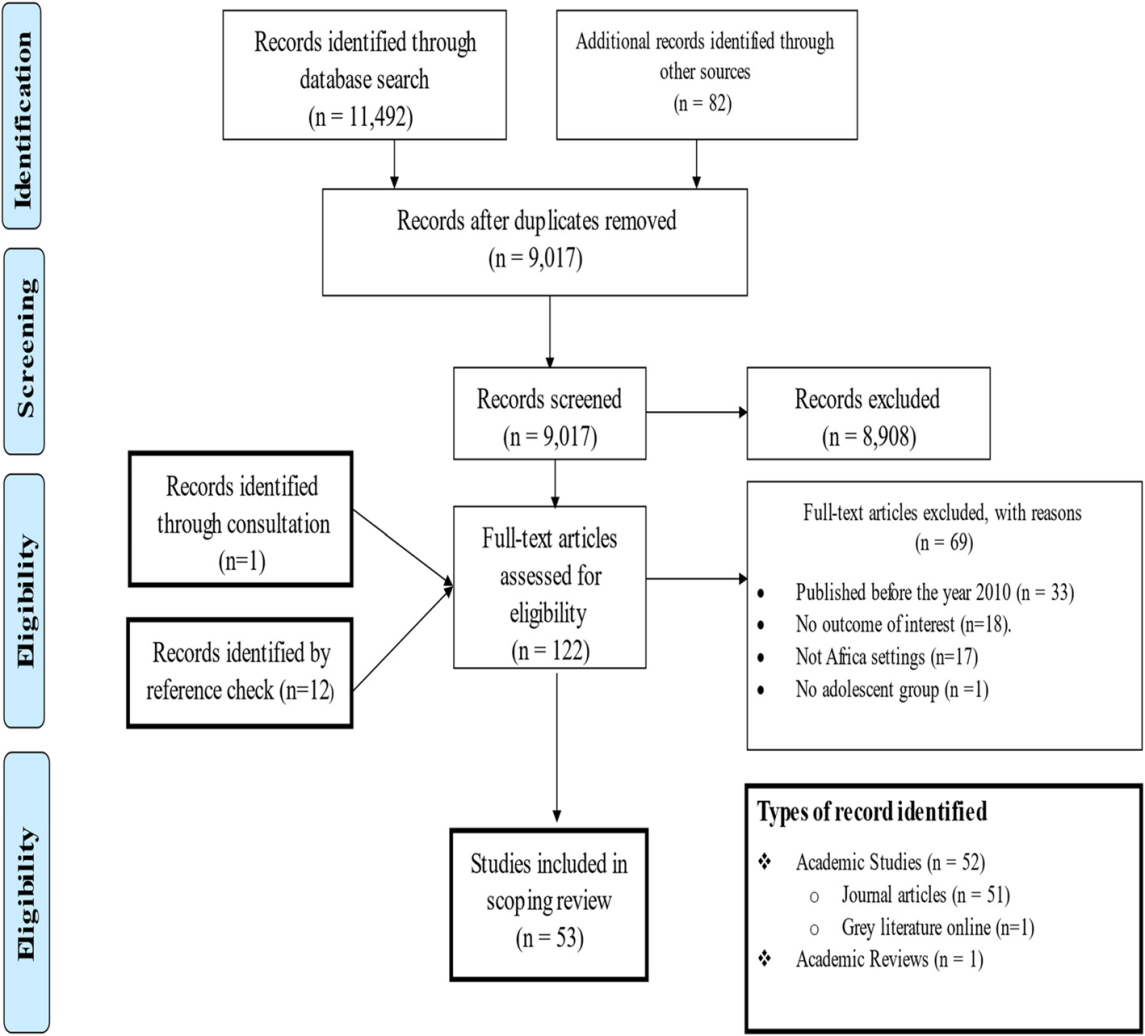
PRISMA flow diagram

We included literature that reported adolescents’ adverse pregnancy outcomes and associated risk factors which were published in English between January 2010 and 31st of August, 2021 from Africa. We included published articles and grey literature [dissertations] and other studies during the screening process. An initial search was carried out in Google Scholar with search terms: “Adolescent pregnancy” OR “adverse outcomes” OR “risk factors” OR “Africa” and yielded 67.800 results. Search terms were refined in January 2021 from academic databases and online search engines (i.e., PubMed Central, Science Direct, JSTOR, The WHO Library, Maternal Surveillance and Response Action Network, Google Scholar, Google, Z-library and Sci-Hub). The last search was done on August 31st, 2021. We also consulted a content expert and a chartered librarian throughout the process of writing this paper. Details of search and screening results are summarized in Table 1 and presented graphically in Fig. 1 using the PRISMA flow diagram.

**Table 1:** Search strategy

Items	Search Strategy
Database	PubMed, CENTRAL, Science direct, JSTOR, The WHO library, Maternal Surveillance and Response Action Network Google Scholar, Google, Z-library and Sci-Hub.
Language filter	2010 and later
Spatial filter	Africa OR Sub-Saharan Africa
Keywords	1. “Adolescents” OR “Adolescent” OR “Adolescence” OR “Teenage” OR “Teen” OR “Teens” OR “Youth” OR “Youths” OR “Girl” OR “Girls”.
	2. “Pregnancy” OR “Childbearing” OR “Pregnancies” OR “Pregnant” OR “Childbirth” OR “Childbirth” OR “Birth” OR “Births”.
	3. “Maternal Mortality” OR “Maternal death” OR “Maternal deaths” OR “Perinatal deaths” OR “Neonatal deaths” OR “Perinatal death” OR “Neonatal death” OR “Low birth Weight” OR “Eclampsia” OR “Preeclampsia” OR “Preterm” OR “Premature” OR “Small-for gestational age” OR “Stillbirth” OR “Premature rapture of membrane” OR “Postpartum hemorrhage” OR “Antepartum hemorrhage” OR “Sepsis” OR “Gestational diabetes” OR “Gestational anemia” OR “Apgar score” OR “Cesarean section” OR “Hospital admission” OR “Fetal infections” OR “Prolonged labor” OR “Fetal distress” OR “Respiratory distress” OR “Pelvic Dystocia” OR “Tearing of tissue” OR “Cephalopelvic disproportion” OR “Pregnancy induced hypertension” OR “NICU admission” OR “Asphyxia” OR “Obstructed labor” OR “Assisted delivery”.
	4. “Risk factors” OR “dangers” OR “Age” OR “Weight” OR “Obesity” OR “ANC attendance” OR “Hospital bookings” OR “Underweight” OR “Diabetes” OR “Malaria” OR “Sexual Transmitted disease” OR “Sexual transmitted infections” OR “HIV/AIDS” OR” HIV” OR “AIDS” OR “High blood pressure” OR “Autoimmune disease” OR “Kidney disease” OR “Asthma” OR “Multiparity” OR “Gravidity” OR “High order pregnancy” OR “Poor nutritional intake” OR “Anemia” OR “Low level of education” OR “Low socioeconomic status” OR “Low health literacy” OR “Rural dwellers” OR “Rural area” OR “Lack of social support” OR “Stigma” OR “Unskilled birth attendants” OR “Long queues at clinics” OR “Attitude of health workers” OR “Abandonment” OR “Child marriage”.
Inclusion criteria	Papers included were: a) Conducted in Africa or Sub-Saharan Africa. b) Conducted on adolescents and reported on adverse pregnancy outcomes and/or associated risks c) peer-reviews articles, literature reviews, dissertation or grey literature. d) Reported in English language e) Conducted 2010 or later.
Exclusion criteria	Papers excluded were: a) Conducted outside Africa b) Full-text articles or conference papers that were not available. c) Studies designed as editorials, letters to editors, commentaries, expert opinions, case reports, and case series. d) Studies that did not provide reports on adolescents’ adverse pregnancy outcomes and/or associated risk factors. e) Studies conducted in a language apart from English. f) Studies completed or published online before 2010.

The first two authors screened the titles together to ensure consistency in approach and independently charted the data, while the third author resolved all discrepancies that occurred. The authors met weekly to discuss issues that resulted from the screening process, disagreements included. Analysis involved thematic and content analysis.

## Results

Of the 53 studies, 51 were journal articles, one was grey literature and one was a review. Fifty-one were quantitative studies, one was a qualitative study, one a mixed-method study and one was a review. Fifty studies sampled 263,580 pregnant women; 46,202 adolescents and 217,378 adults (> 20 years;). One study sampled the live births of 68,191 adolescents and 215,199 adults. Additional file 1: Table 2 summarizes the characteristics of the included studies. Details of the data are presented in Additional file 1: Table 2, Tables 2, 3 and 4.

**Table 2:** Adverse obstetric outcomes associated with adolescent pregnancy

Pregnancy outcome	Age groups	Study
Preeclampsia/ eclampsia	10–19 years	Mezmur et al., 2021 [17]; Tshakweni et al., 2020 [18]; Grønvik et al., 2018 [7]; Abdelsattar et al., 2016 [19]; Edessy et al., 2015 [20]; Ijarotimi et al., 2014 [21]; Adeyinka et al., 2010 [22]; Zeck et al., 2010 [23]; Abebe et al., 2020 [24]; Alyamani et al., 2021 [25]; Gueye et al., 2020 [26]
	10–15 years	Moraes et al., 2018 [27]; Rasheed et al., 2011 [28]; Govender et al., 2018 [29]
	20–45 years	Mezmur et al., 2021 [17]; Tembo et al., 2020 [30]
Maternal death	10–19 years	November et al., 2018 [31]; de Wet, 2016 [32]; Ijarotimi et al., 2014 [21]; Grønvik et al., 2018 [7]; Ganchimeg et al., 2013 [33]; Alyamani et al., 2021 [25]
Cesarean Section	10–19 years	Yussif et al., 2017 [34]; Edessy et al., 2015 [20]; Ijarotimi et al., 2014 [21]; Iklaki et al., 2012 [35]; Ayuba et al., 2012 [36]; Tebeu et al., 2011 [37]; Zeck et al., 2010 [23].
	10–15 years	Ganchimeg et al., 2013 [33]; Rasheed et al., 2011 [28]
	20–45 years	Alyamani et al., 2021 [25]; Hussein Eldessouki et al., 2020 [38]; Abebe et al., 2020 [24]; Abbas et al., 2017 [39]; Abdelsattar et al., 2016 [19]; Hoque et al., 2014 [40]; Muganyizi et al., 2013 [41]; Omole_Ohonsi et al., 2010 [42]; Hoque et al., 2010 [43]
Obstetric hemorrhage	10–19 years	Gyimah et al., 2020 [44]; Tembo et al., 2020 [30]; Ijarotimi et al., 2014 [21]; Ayuba et al., 2012 [36]; Hussein Eldessouki et al., 2020 [38]
	10–15 years	Moraes et al., 2018 [27]
Obstructed labor	10–19 years	Tembo et al., 2020 [30]; Tshakweni et al., 2020 [18]; Ijarotimi et al., 2014 [21]
PROM	10–19 years	Abdelsattar et al., 2016 [19]; Tembo et al., 2020 [30]
	10–15 years	Rasheed et al., 2011 [28]
ICU admission/ hospital admission	10–19 years	Alyamani et al., 2021 [25]
Gestational hypertension	10–19 years	Edessy et al., 2015 [20]
	20–45 years	Alyamani et al., 2021 [25]; Tembo et al., 2020 [30]; Abdelsattar et al., 2016 [19]
Gestational diabetes	10–19 years	Abdelsattar et al., 2016 [19]; Alyamani et al., 2021 [25]
Gestational anemia	10–19 years	Tshakweni et al., 2020 [18]; Ijarotimi et al., 2014 [21]; Ezegwui et al., 2012 [45]
	10–15 years	Govender et al., 2018 [29]
Cephalopelvic disproportion	10–19 years	Gueye et al., 2020 [26]; Mubikayi, 2020 [46]
	10–15 years	Moraes et al., 2018 [27]; Ganchimeng et al., 2013 [33]
Prolonged labor	10–19 years	Tshakweni et al., 2020 [18]
	10–15 years	Moraes et al., 2018 [27]
Assisted vaginal birth	10–19 years	Ezegwui et al., 2012 [45]; Zeck et al., 2010 [23]
	20–45 years	Ayuba et al., 2012 [36]; Omole_Ohonsi et al., 2010 [42]
Emotional vulnerability	10–15 years	Govender et al., 2018 [29]

**Table 3:** Adverse perinatal/neonatal outcomes associated with adolescence pregnancy

Pregnancy outcome	Age groups	Study
LBW	10–19 years	Mezmur et al., 2021 [17]; Serunjogi et al., 2021 [47]; Pons-Duran et al., 2021 [48]; Gyimah et al., 2020 [44]; Siakwa et al. 2020 [49]; Abebe et al., 2020 [24]; Kamala et al., 2018 [50]; Gronvik et al., 2018 [7]; Agbor et al., 2017 [51]; Njim et al., 2017 [52]; Abbas et al., 2017 [39]; Abdelsatter et al., 2016 [19]; Njim et al., 2016 [53]; Schipulle 2015 [54]; Ngowa et al., 2015 [55]; Muganyizi et al., 2013 [41]; Ezegwui et al., 2012 [45]; Adeyinka et al., 2010 [22]; Kurth et al., 2010 [56]; Omole- Ohonsi et al., 2010 [42]; Zeck et al., 2010 [23]; Hussein Eldessouki et al., 2020 [38]; Kassa et al., 2019 [57]; Bihoun et al., 2017 [58]; Egbe et al., 2015 [59]; Granchimeng et al., 2013 [33]
	10–15 years	Moraes et al., 2018 [27]; Govender et al., 2018 [29]; Mombo-Ngoma et al., 2016 [60]
	35+ years	Kamala et al., 2018 [50]; Muganyizi et al., 2013 [41]
Preterm birth	10–19 years	Mezmur et al., 2021 [17]; Serunjogi et al., 2021 [47]; Pons-Duran et al., 2021 [48]; Gyimah et al., 2020 [44]; Andemel et al., 2020 [61]; Abebe et al., 2020 [24]; Grønvik et al., 2018 [7]; Njim et al., 2016 [53]; Ngowa et al., 2015 [55]; Edessy et al., 2015 [20]; Fouelifack et al., 2014 [62]; Ezegwui et al., 2012 [45]; Ayuba et al., 2012 [36]; Omole- Ohonsi et al., 201 0[42]; Gueye et al., 2020 [26]; Hussein Eldessouki et al., 2020 [38]; Kassa et al., 2019 [57]; Bihoun et al., 2017 [58]; Egbe et al., 2015 [59]; Granchimeng et al., 2013 [33]
	10–15 years	Mombo-Ngoma et al., 2016 [60]; Rasheed et al., 2011 [28]; Tebeu et al., 2011 [37]
	20–45 years	Siakwa et al., 2020 [49]
Neonatal death	10-19 years	Serunjogi et al., 2021 [47]; Gyimah et al., 2020 [44]; Noveber et al., 2018 [31] Yussif et al., 2017 [34]; de Wet, 2016 [32]; Ganchimeng et al., 2013 [33]
	10-15 years	Moraes et al., 2018 [27]; Neal et al., 2018 [10]; Ijarotimi et al., 2014 [21]
Perinatal deaths	10-19 years	Andemel et al., 2020 [61]; Kamala et al., 2018 [50]; Grønvik et al., 2018 [7]; Ezegwui et al., 2012 [45]; Laari et al., 2016 [63]
	35+ years	Kamala et al., 2018 [50]
Stillbirth	10–19 years	Kamala et al., 2018 [50]; Yussif et al., 2017 [34]; Edessy et al., 2015 [20]; Iklaki et al., 2012 [35]; Tebeu et al., 2011 [37]; Adeyinka et al., 2010 [22]; Hussein Eldessouki et al., 2020 [38]; Fouelifack et al., 2014 [62]; Zeck et al., 2010 [23]
	35+ years	Kamala et al., 2018 [50]; Muganyizi et al., 2013 [41]
Low Apgar score	10–19 years	Mezmur et al., 2021 [17]; Tshakweni et al., 2020 [18]; Abbas et al., 2017 [39]; Kamala et al., 2018 [50]; Hogue et al., 2014 [40]; Muganyizi et al., 2013 [41]; Ezegwui et al., 2012 [45]; Adeyinka et al., 2010 [22]
	10–15 years	Moraes et al., 2018 [27]; Govender et al., 2018 [29]
	20–45 years	Siakwa et al., 2020 [49]
	35+ years	Kamala et al., 2018 [50]
Small-for-gestational age	10–19 years	Pons-Duran et al., 2021 [48]
	10–15 years	Govender et al., 2018 [29]
Neonatal respiratory distress	10–19 years	Jaen-Sanchez et al., 2020 [64]; Gueye et al., 2020 [26]
Fetal/perinatal distress	10–19 years	Tshakweni et al., 2020 [18]; Fouelifack et al., 2014 [62]
Fetal infections	10–19 years	Gueye et al., 2020 [26]
Birth asphyxia	10–19 years	Agbor et al., 2017 [51]; Njim et al., 2016 [53]; Ijarotimi et al., 2014 [21]; Gueye et al., 2020 [26]
	10–15 years	Moraes et al., 2018 [27]
NICU admission	10–19 years	Abdelsattar et al., 2016 [19]
Congenital disorders	10–19 years	Edessy et al., 2015 [20]

**Table 4:** Risk factors associated adolescence poor pregnancy outcomes

Major theme	Sub-theme	Study
Socio-demographic factors	Poor socioeconomic status	Alyamani et al., 2021 [25]; Mezmur et al., 2021 [17]; Hussein Eldessouki et al., 2020 [38]; Noveber et al., 2018 [31]; Govender et al., 2018 [29]; Abbas et al., 2017 [39]; Egbe et al., 2015 [59]; Ijarotimi et al., 2014 [21]; Ganchimeg et al., 2013 [33]; Ezegwui et al., 2012 [45]; Ayuba et al., 2012 [36]; Zeck et al., 2010 [23]
	Low education	Hussein Eldessouki et al., 2020 [38]; Kamala et al., 2018 [50]; Abbas et al., 2017 [39]; Rasheed et al., 2011 [28]; Zeck et al., 2010 [23]
	Low health literacy	November et al., 2018 [31]; Zeck et al., 2010 [23]
	Married	Agbor et al., 2017 [51]; Abdelsatter et al., 2016 [19]
	Not married	Kassa et al., 2019 [57]; Fouelifack et al., 2014 [62]; Ayyuba et al., 2012 [36]; Egbe et al., 2015 [59]
	Age [10–16]	Moraes et al., 2018 [27]; neal et al., 2018 [10]; Mombo-Ngoma et al., 2016 [60]; Rasheed et al., 2011 [28]; Kurth et al., 2010 [56]
	Age [< 20]	Andemel et al., 2020 [61]; Abebe et al., 2020 [24]; Mubikayi, 2020 [46]; Kamala et al., 2018 [50]; Grønvik et al., 2018 [7]; Bihoun et al., 2017 [58]; Yussif et al., 2017 [34]; Abbas et al., 2017 [39]; Egbe et al., 2015 [59]; Ngowa et al., 2015 [55]; Edessy et al., 2015 [20]; Fouelifack et al., 2014 [62]; Ijarotimi et al., 2014 [21]; Ayuba et al., 2012 [36]; Tebeu et al., 2011 [37]; Adeyinka et al., 2010 [22].
	Rural area	Hussein Eldessouki et al., 2020 [38]; Kassa et al., 2019 [57]; Agbor et al., 2017 [51]; Bihoun et al., 2017 [58]; Abbas et al., 2017 [39]; Laari et al., 2016 [63]; Abdelsatter et al., 2016 [19]; Edessy et al., 2015 [20]; Rasheed et al., 2011 [28].
Risky behaviour/ attitudes	No/low/late ANC attendance	Alyamani et al., 2021 [25]; Mezmur et al., 2021 [17]; Jean-Sanchez et al., 2020 [64]; Gueye et al., 2020 [26]; Tshakweni et al., 2020 [18]; Abebe et al., 2020 [24]; Kassa et al., 2019 [57]; Kamala et al., 2018 [50]; Govender et al., 2018 [29]; Laari et al., 2016 [63]; Schipulle, 2015 [54]; Edessy et al., 2015 [20]; Ijarotimi et al., 2014 [21]; Muganyizi 2013 [41]; Ganchimeng et al., 2013 [33]; Iklaki et al., 2012 [35]; Kurth et al., 2010 [56]; Omole-Ohonsi et al., 201 0[42]
	Delayed care seeking	November et al., 2018 [31]
	Inadequate nutrition	Gyimah et al., 2020 [44]; Bihoun et al., 2017 [58]; Neal et al., 2018 [10]
	Alcohol intake	Laari et al., 2016 [63]
	No facility birth	Omole-Ohonsi et al., 201 0[42]
Pregnancy characteristics	Subsequent births	Neal et al., 2018 [10]
	Multiparity	Kamala et al., 2018 [50]
	Gravidity	Egbe et al., 2015 [59]; Ibrahim et al., 2015 [65]
	High order pregnancy	Obare et al., 2012 [66]
Health facility factors	poor ANC quality	Chaibva et al., 2019 [67]; Omole-Ohonsi et al., 2010 [42]
	Long queues	Govender et al., 2018 [29]
	Attitude of health workers	Govender et al., 2018 [29]
	Long distance	Govender et al., 2018 [29]
	Stigma	November et al., 2018 [31]
Adolescent pregnancy-related Stigma and abuse	Stigma	November et al., 2018 [31]
	Abandonment	Omole-Ohonsi et al., 2010 [42]
	Lack of family and social support	Omel-Ohonsi et al., 2010 [42]
	IPV	Mezmur et al., 2021 [17]
Previous obstetric complications	Previous abortion	Jean-Sanchez et al., 2020 [64]; Abass et al., 2017 [39]; de Wet, 2016 [32]; Edessy et al., 2015 [20]
	Previous CS	Abbas et al, 2017 [39]
Diseases/infections	HIV	Jean-Sanchez et al., 2020 [64]; Govender et al., 2018 [29]; Obare et al., 2012 [66]
	Malaria	Mombo-Ngoma et al., 2016 [60]
	Hypertension	de Wet, 2016 [32]

### Adverse obstetric and perinatal/neonatal outcomes in adolescent pregnancy

To address the first research question, two major themes were generated: (1) adverse obstetric outcomes in adolescent pregnancies and (2) adverse perinatal or neonatal outcomes in adolescent pregnancies.

#### Adverse obstetric outcomes in adolescent pregnancies

Regardless of the study design, we found a high prevalence of negative obstetric outcomes among adolescent mothers (Table 2). Preeclampsia and eclampsia were found to be more common in pregnant adolescents than in adults [7, 17/27]. Studies focusing on adolescent sub-group analysis found that young adolescents had a higher incidence of preeclampsia and eclampsia than older adolescents and adults [27, 28]. Moreover, maternal mortality is higher among adolescent than among adult mothers [7, 21, 25, 31–33]. One study compared adolescent mothers and advanced age mothers (> 34 years) and found a higher incidence of maternal deaths in the latter [41]. More studies found higher incidences of cesarean birth in adults than in adolescents [19–21, 23–25, 34–43]. Contrarily, two other studies reported a higher incidence of cesarean birth in young adolescents than in older adolescents and adults [28, 33].

Evidence also indicated that adolescents had higher rates of postpartum and antepartum hemorrhage than adults [17]. Obstructed labor, premature rupture of membranes (PROM) and gestational diabetes are also more common among adolescents [18, 19, 21, 25, 27]. Rasheed et al. [28] observed that younger adolescents are more vulnerable to PROM than their older counterparts. It was also revealed that adolescent mothers are more likely than adult mothers to be admitted to intensive care units (ICU) [25, 45]. Furthermore, adolescent mothers had a higher incidence of gestational anemia, prolonged labor and cephalopelvic disproportion than adult mothers [18, 21, 26, 28, 47–49]. Moreover, assisted vaginal births were similar for adolescent and adult mothers [23, 29, 36, 42]. Again, cephalopelvic disproportion [27, 33] and emotional trauma [46] are more common in young adolescent girls [28, 32, 48]. Adults, however, had a higher prevalence of gestational hypertension than adolescents [19, 20, 25, 30].

#### Adverse perinatal and neonatal outcomes in adolescent pregnancies

We found an increased risk of adverse perinatal and neonatal outcomes for adolescent mothers (Table 3). Adolescent mothers are at higher risk of LBW and preterm births and younger adolescents are at higher risk of LBW with preterm births than their older counterparts [7, 17, 19, 20, 22–24, 26, 28, 29, 32–35, 38, 42, 44–48, 50–63, 65]. The review also showed that the two extreme age groups in pregnant women (below age 15 and above age 40) have a higher incidence of LBW compared with older adolescents [41, 50]. One study, however, found that preterm births are more prevalent in adult mothers compared to adolescents [49].

Studies that compared adolescents to adults have also showed higher risks of neonatal, perinatal and fetal deaths and stillbirths among adolescent mothers [7, 20, 22, 23, 30–32, 34, 39, 41, 44, 45, 47, 50, 52, 62, 63, 66]. Within adolescent groups, some studies reported that young adolescents are at a greater risk of neonatal mortality, while advanced age mothers are at higher risks of perinatal deaths [50] and stillbirths [41, 50] compared to adolescents [10, 21, 28, 33, 52]. Low Apgar scores were more common among adolescents than in adults [17, 18, 22, 29, 39–41, 49, 50]. Meanwhile, other studies have found that low Apgar scores are more common in young adolescents than in older adolescents [27, 46]. According to Kamala et al., low Apgar scores are more common among advanced-age mothers than in adolescents [52]. Newborns from advanced age mothers are also more likely small-for-gestational-age, to have neonatal respiratory distress, fetal or perinatal distress, fetal infections, birth asphyxia, neonatal intensive care units (NICU) admission and congenital disorders [18–21, 26, 45, 51, 53, 62, 64]. However, two studies reported that newborns from younger adolescents are also more likely small-for-gestational-age and to have birth asphyxia [27, 46].

### Risk factors associated with adverse pregnancy outcomes among adolescents

Five major themes were generated about risk factors that expose adolescent pregnancies to adverse outcomes. Socioeconomic factors, risky behaviors or lifestyle, pregnancy characteristics, health facility factors, adolescent pregnancy-related stigma and abuse, previous obstetric complications, disease and infections are important here (Table 4).

#### Socio-economic factors

Age 10 to 16 years and less than 20 years were identified as major risk factors [7, 10, 20, 21, 24, 28, 29, 32, 33, 35, 38, 39, 41, 49, 52, 53, 58, 59, 61–63, 65]. Some studies revealed that adolescents with low socioeconomic status, low education and low health literacy are at higher risks of maternal and neonatal mortality and morbidities [17, 21, 23, 25, 29, 30, 32, 34, 35, 42, 47, 48, 52, 53]. Moreover, adolescents in rural areas and those who are not married are at risk of having poor pregnancy outcomes [19, 20, 29, 34, 35, 42, 53, 54, 60, 61, 63, 66]. Married adolescents, however, are not immune to unfavorable pregnancy outcomes [19, 51].

#### Risky behaviors or lifestyle

Pregnant adolescents who have no, few or late appointments for antenatal care (ANC) are more likely to have negative pregnancy outcomes due to delayed care-seeking and a preference for unskilled birth [17, 18, 20, 21, 24–26, 30, 32, 33, 38, 41, 48, 52, 57, 59, 60, 66, 67]. Meanwhile, it is revealed that lifestyle choices such as alcohol consumption and inadequate nutrition intake put pregnant adolescents at higher risk of pregnancy complications [10, 45, 61, 66].

#### Health facility factors and pregnancy characteristics

Some factors of health facilities also discourage adolescents from seeking and receiving appropriate care. Adolescents are discouraged from seeking maternal health care because of poor ANC quality, long queues and waiting time, negative attitudes of health care workers, long distances to health centers and stigma directed towards pregnant adolescents by health care professionals [31, 42, 46, 67]. Moreover, adolescents who are multiparous or have a high order pregnancy are at higher risks of adverse pregnancy outcomes [10, 40, 52, 53, 64].

#### Stigma and abuse

Adolescents who are pregnant face stigma and abuse from their peers and family members [31, 42]. Rejection from family members deprives them of necessary family and social support [42]. They may even face partner violence which puts them and the fetus at risk [17].

#### Previous obstetric complications, disease and infections

Adolescents with a history of abortion and cesarean birth are at higher risks of adverse pregnancy outcomes [20, 32, 39, 64]. Infections and diseases such as HIV/AIDS, malaria and hypertension put pregnant adolescents at risk of having poor pregnancy outcomes [32, 46, 60, 64, 66].

## Discussion

Our findings revealed that adolescent pregnancy and childbirth are linked to poor maternal and perinatal health outcomes. Many maternal morbidities, emotional trauma included, are more common in adolescent mothers in Africa. The same holds true for perinatal mortality and morbidity. Younger adolescents [≤15 years] have higher risks of negative pregnancy outcomes than older adolescents [16–19 years] and adult mothers. In addition to maternal mortality, adolescent mothers may face other health risks in the future. The International Diabetes Federation (IDF) observed that among women with gestational diabetes, 5.7% develop Type 1 diabetes within 7 years and 50.4% develop Type 2 diabetes within 23 years [68]. High adolescent fertility, as well as high adverse pregnancy outcomes in the African continent may explain why sub-Saharan Africa had the highest neonatal mortality rates per 1000 live births in 2019 [1, 30]. It is also clear that the majority of perinatal and neonatal deaths in 2017 were caused by preterm birth, LBW, birth asphyxia and sepsis [69]. Can Africa achieve SDG 3 by the year 2030 with these high numbers of complicated adolescent pregnancies?

Adolescent pregnancy is of much concern because adverse pregnancy outcomes are associated with a variety of vulnerabilities such as child marriage, female genital mutilation (FGM) and unsafe abortion [2]. Young adolescents may be physiologically immature and at high risk of nutritional deficiencies and hypertension [10]. Thus, African governments and their health agencies would need to prevent adolescents from becoming pregnant and be physiologically mature enough, when they start with childbearing.

### Risk factors affecting poor adolescent pregnancy outcomes in Africa

Our review indicated that adolescents in Africa, regardless of their marital, socioeconomic, educational and health literacy status, are at risk of poor pregnancy outcomes. Risky behaviors or attitudes, such as delayed health care seeking, insufficient nutrition intake, birth at home and alcohol consumption put pregnant adolescents at risk of poor pregnancy outcomes. Moreover, poor ANC quality, poor attitudes of health care professionals, stigma and abuse are risk factors that expose adolescents to poor pregnancy outcomes. Pregnant adolescents in Africa are highly susceptible to highly prevalent infections on the continent that affect birthweight and gestational age at birth [10]. Adolescents have a 20–40% risk of vertical transmission of HIV and other STIs to the fetus.

Pregnant adolescents face rejection, stigma and abuse from family members and intimate partners, which may have both direct and indirect impacts on their pregnancy outcomes [17, 42]. Moreover, with low levels of health literacy, adolescents are likely to face difficulties to access health care, understand health information and effectively use such information to maintain and promote healthy pregnancy outcomes [2, 23, 31]. Pregnant adolescent girls in low-resource settings have negative attitudes toward seeking maternal health care because of highly fragmented, poorly coordinated, low quality of care [2, 7]. Pregnant teenagers regard primary health care as inaccessible for fear of stigma, lack of respect, privacy and confidentiality, discrimination and imposition of moral norms, particularly in resource-limited settings such as Africa [2, 18, 25]. In most African countries, particularly in rural areas, poor health and road infrastructure, as well as lack of health care professionals may also prevent pregnant adolescents from seeking quality health care.

Child marriage is highly prevalent in Africa and has been linked to poor maternal outcomes, intimate partner violence (IPV’, limited decision to seek health care during pregnancy and maternal mortality [70]. A married girl may have little or no ideas about contraception and may lack the decision authority to even time and space pregnancy. Meanwhile, poorly timed or unwanted pregnancies are often associated with a high risk of morbidity and mortality in adolescents, because of high numbers of abortions [71]. Abortion, however, is highly stigmatized in Africa and adolescents are forced to have unsafe abortions due to lack of care, high costs, stigma, refusal by health care workers, needless requirements and morality [1]. In Ghana, for example, unsafe abortions account for 11–30% of maternal deaths, with adolescents accounting for 35% of these [71]. The phenomenon of adolescent pregnancy and its related adverse pregnancy outcomes are multifaceted and interdisciplinary issues, and the earlier evidence-based proactive measures are put in place, the better.

### Policy implications

Eliminating adolescent pregnancy will significantly reduce maternal and perinatal mortality, and accelerate progress toward achieving the maternal and perinatal components of the SDGs. We propose not only reducing adolescent births, but also reducing births in the most vulnerable girls, such as those under the age of 16, those living on the streets and in rural areas in displaced settlements in Africa. In Africa, particularly in sub-Saharan Africa, there has been minimal progress in reducing adolescent births, which calls for improvement in young girls’ health by preventing adolescent pregnancy [10].

To be successful in preventing adolescent pregnancy, efforts are needed to eliminate child marriage, poverty and all forms of abuse and stigma directed at adolescent girls, particularly during pregnancy when they attempt to seek quality health care. Adolescents need to have access to abortion and post-abortion care in Africa. Moreover, significant efforts are needed to improve sexual and reproductive health by developing appropriate approaches and strategies to meet the needs of adolescents. All vulnerable and at-risk girls such as homeless, out of school, living in urban slums or displaced, need to be provided with comprehensive, culturally and socially appropriate sexual education as well as essential and adolescent-friendly sexual and reproductive health care.

Pregnant adolescents will have better pregnancy outcomes if they have access to adequate and high-quality ANC and postnatal care. This package of maternal and child care could include nutritional programs, treatments for infection and prompt attention to treatable obstetric conditions. All impediments to pregnant adolescents’ access to health care seeking need to be removed.

### Recommendations for future research

Few studies in this review attempted to control for socio-demographic factors. Because the majority were retrospective records´ reviews, some relevant socioeconomic factors such as educational attainment and income status were missing. Future research needs to also prioritize high-quality studies of younger as compared with older adolescents rather than grouping them together. Some studies had small sample sizes drawn from hospital settings, which may exclude more vulnerable groups and have an impact on generalization. There is a high likelihood that pregnant adolescents in Africa will give birth outside a health care setting, and future studies need to include this population to help improve generalization and estimation of adverse pregnancy outcomes and risk factors. Future studies focusing on medical records may avoid information bias, but they will also face incomplete and insufficient medical records.

### Limitations

There is a possibility of publication bias, because negative results are unlikely to be published. Studies were also restricted to those published in English, which may have an impact on outcomes of this review. Despite this limitation, this review compiled relevant studies, including a large sample, from four major databases, five other online sources and 21 nations in Africa. Furthermore, the authors sought advice from experts who were deemed relevant for this review to provide comprehensive evidence on adolescent pregnancy and associated adverse pregnancy outcomes in Africa.

## Conclusion

Adolescent pregnancy is associated with a higher risk of adverse pregnancy outcomes. Aside age, factors that put pregnant adolescents at high risk of poor pregnancy outcomes include low socioeconomic and educational status, poor utilization of ANC, risky lifestyles such as alcohol consumption, and unappealing health care factors. Maternal health utilization was identified as an important factor in improving pregnancy outcomes among adolescents in Africa. Aside increased efforts to prevent adolescent pregnancy, it is also prudent to increase adolescent utilization of maternal health care, as well as address issues of stigma, abortion and child marriage in Africa. There is a need for high-quality observational studies on young adolescents that account for confounders in Africa.

## Supplementary Information

**Additional file 1: Table 2.** Extracted data and study characteristics.

## Data Availability

All data generated or analyzed during this study are included in this published article (and its supplementary information files).

## Abbreviations

HIV: Human Immunodeficiency Virus
AIDS: Acquired Immunodeficiency Syndrome
LBW: Low Birth Weight
WHO: World Health Organization
PROM: Premature Rupture of Membranes
IDF: International Diabetes Federation
NICU: Neonatal Intensive Care Unit
ANC: Antenatal Care
PNC: Postnatal Care
ICU: Intensive Care Unit
IPV: Intimate Partner Violence
