# Supplementary material for: Adverse obstetric and neonatal outcomes of adolescent pregnancies in Africa: a scoping review
Source: BMC Pregnancy Childbirth. 2022 Jul 27;22:598. doi: 10.1186/s12884-022-04821-w (PMC9327294; doi:10.1186/s12884-022-04821-w)
Supplement: Supplementary file 1 — Additional file 1: Table 2. Extracted data and study characteristics. [file 12884_2022_4821_MOESM1_ESM.docx]

**Table 2: Extracted data and study characteristics**

| **Author[s], Year and Location** | **Aims/Purpose of the study** | **Method** | **Study Population/ Sample size/** | **Main outcome/conclusion** |
| --- | --- | --- | --- | --- |
|  |  |  |  |  |
| Alyamani et al, 2021  Egypt | To find out the prevalence of teenage pregnancy and its maternal and fetal outcome in comparison. | Quantitative; Case control study | Pregnant women:  16 - 19y = 538  25 – 29y = 609 | Teenager primigravid pregnancy should be considered as high-risk pregnancy and thus require special medical attention to avoid adverse maternal and neonatal outcomes |
| Mezmur et al, 2021  Ethiopia | To compare fetal outcomes between teen and adult pregnant women from rural Eastern Ethiopia | Quantitative; Cross sectional survey | Pregnant women:  13 – 19y = 481  20 – 34y = 481 | Teenage pregnancy is associated with a high rate of adverse fetal outcomes |
| [Serunjogi et al., 2021]  Uganda | To compare the risk of adverse birth outcomes between adolescent and adult mothers. | Quantitative;  Case-control study | Pregnant women:  12-19y = 11,028  20-34y = 89,161 | This study found that adolescent mothers had an increased risk for several adverse birth outcomes compared to adult mothers. |
| [Pons-Duran et al., 2021]  Mozambique | To evaluate the association between age, HIV infection and their interaction and the risk of maternal morbidity and adverse pregnancy and perinatal outcomes. | Quantitative;  Secondary analysis of data | Pregnant women:  ≤ 19 = 729  20 – 24 =628  ≥ 25 995 | Adolescence was associated with an increased risk of poor morbidity, pregnancy and perinatal outcomes, irrespective of HIV infection. |
| [Gueye et al., 2020]  Senegal | To assess morbidity and analyze the causes of neonatal mortality linked to these early pregnancies. | Quantitative; Descriptive survey | Pregnant women:  14 – 19y = 209 | Teenage pregnancy is the source of many complications, especially in newborns. It is necessary to conduct communications campaigns in order to stem them. |
| [Gyimah et al., 2020]  Ghana | To examined the relationship between nutritional status and birth outcomes among Ghanaian teenagers | Quantitative: Prospective Cohort study | Pregnant women:  13-19y = 416 | Interventions for improving the nutritional status of teenagers before and during pregnancy are urgently needed to reduce the risk of adverse birth outcomes |
| Hussein Eldessouki et al, 2020  Egypt | To examine the association between young age pregnancy and increased risk of adverse pregnancy outcome and also compare pregnancy outcome of adolescents and adults. | Quantitative; Descriptive study | Pregnant women:  15 – 19y = 125  20 – 29y = 490 | Pregnancy before age 20 is a high-risk pregnancy and leads to unfavorable pregnancy outcome and has a negative impact on maternal and neonatal health. |
| Andemel et al, 2020  Mali | To obtain background information on poor pregnancy outcomes among adolescents | Quantitative | Pregnant women:  < 20y =538  20 -35y =1172  >35y =104 | Young age predicts perinatal deaths and preterm. |
| Tembo et al, 2020  Zambia | To explore associations between maternal age and neonatal outcomes among women. | Quantitative;  Prospective cohort study | Pregnant women:  10 – 19 y = 1788  20 – 24y = 3980  25 – 34y = 4651  35+y = 1082 | Adolescence did not have statistically significant associations with poor maternal health outcomes. However, the risk of experiencing obstructed labour, premature rupture of membranes and postpartum hemorrhage was higher among adolescents. |
| [Tshakweni et al., 2020]  South Africa | To examine maternal and neonatal adverse outcome among teenagers compared to older pregnant women. | Quantitative;  Retrospective study | Pregnant women:  13-19y = 95  20-29y =51  30-39y =42  40+y =8 | The findings of this study revealed that the teenagers start booking at the second trimester, which may impose the risk of complications if not observed at an early stage. |
| Siakwa et al., 2020  Ghana | To compare maternal and perinatal outcomes among teen and adult mothers. | Quantitative;  Case-control observational study | Pregnant women:  13-19y = 503  20 – 34y = 503 | Prevalence on adverse outcomes was low. Improved access to prenatal care utilization would produce better pregnancy outcomes. |
| Jaen-Sanchez et al., 2020  Mozambique | To identify the factors associated with adolescent motherhood | Quantitative;  Cross-sectional study | Pregnant women:  13-19y = 255  20-45y = 566 | Teenage motherhood is a serious public health problem in Mozambique. Intensive sexual and reproductive health planning for adolescents is needed. |
| Abebe et al., 2020  Ethiopia | To determine adverse obstetrical and perinatal outcomes of teenage pregnancy. | Quantitative;  Retrospective cohort study | Pregnant women:  13-18y = 309  19-27y = 309 | Adverse obstetric and perinatal outcomes were significantly more frequent associated with teenagers than adult mothers. ANC utilization is a key factor. |
| Mubikayi, 2020  DR Congo | To identify the epidemiologic profile of the teenage mothers and related obstetric issues. | Quantitative; Retrospective descriptive study | Pregnant women:  14-16y = 6  17-19y = 61 | Teenage pregnancy is a risk factor for childbirth and educating young girls about family planning is pertinent. |
| **Table 2: Extracted data and study characteristics (Continued)** | | | | |
| Kassa et al., 2019  Ethiopia | To assess the adverse national outcomes of adolescent pregnancy | Quantitative;  Case control study | Pregnant women:  15-19y = 374  20-34y = 760 | Adolescent women were less likely to receive ANC. Babies born from adolescent mothers are at higher odds of adverse neonatal outcomes. |
| Chaibva et al., 2019  Zimbabwe | To describe the prevalence and factors contributing to adverse pregnancy outcomes | Quantitative | Pregnant women:  <20y = 47  20-34y = 247  >34y = 47 | High prevalence of adverse pregnancy outcomes in Mutare district could be reduced through the provision of quality antenatal care throughout the continuum of care, pre-, intra and postpartum. |
| Moraes et al., 2018  Zambia | To investigate obstetric and perinatal outcomes among adolescents compared to mothers aged 20-24 years. | Quantitative;  Retrospective descriptive study | Pregnant women:  10-15y = 81  16-17y = 396  18 – 19y = 814  20 – 24y = 1504 | The findings demonstrate that adolescent pregnancy increases the risk of adverse obstetric and perinatal outcomes. Adolescent pregnancy, especially in mothers younger than 16 years, increases the risk of adverse obstetric and perinatal outcomes |
| Neal et al., 2018  LMIC [SSA] | To explore the impact of early motherhood on neonatal mortality and assess whether the risk of neonatal mortality is greater for younger adolescent mothers compared to mothers in late adolescence. | Quantitative | Number of births:  <16y = 7163  16-17y = 23852  18-19y = 37176  20-29y = 215199 | The findings indicate there is an increase in risk of neonatal deaths in adolescent mothers, with the risk most marked in youngest age group. The increased risk associated with adolescent birth was greater for second or subsequent birth. |
| Kamala et al., 2018  Tanzania | To determine the prevalence, predictors and perinatal outcomes of LBW | Quantitative;  Retrospective study | Pregnant women:  <20y = 2150  20-35y = 31095  >35y = 5005 | Future interventions should focus on improving the quality of ANC and integrating peripartum emergency obstetric and neonatal care. |
| Govender et al., 2018  South Africa | To evaluate maternal and birth outcomes related to these pregnancies and to identify factors influencing access to ANC among pregnant adolescents. | Mixed-method | Pregnant women:  13-16y = 314 | The study showed that adolescent pregnancy in this research setting was associated with a risk of late booking and reduced ANC visits, which could lead to adverse maternal and birth outcomes. |
| Grønvik et al., 2018  SSA | To examine whether childbearing before age 18 in Sub-Saharan Africa is associated with increased risk of maternal and child complications. | Review | Pregnant women:  <18y  20 – 35y | The findings indicate that young maternal age is associated with same unfavorable outcomes in SSA. High quality observational studies that adjust for socioeconomic factors are lacking. |
| November et al., 2018  Liberia | To explore the causes of high incidence of maternal mortality in young teenagers and identify possible interventions to improve. | Qualitative study | Population:  Adolescents = 11  Men [<29] = 9  Midwives = 25  Headteachers =2  Consultant advisors to UNICEF/UNFPA =2  Advisors at MOH =2  Advisors at MSW =1  Project lead NGOs =2  Manager of CBO =1 | For girls who do become pregnant, risks are believed to be related more to stigma and abandonment than to physical maturity, leading to lack of family-based support and delayed care-seeking for antenatal and childbirth care. |
| Agbor et al., 2017  Cameroon | To evaluate the trend and the adverse maternofetal outcomes of adolescent births. | Quantitative;  Retrospective study. | Pregnant women:  <20y = 368  >19y = 1435 | Adolescent mothers are more likely to sustain second - fourth degree perineal tears during labour and their babies have a higher tendency of being asphyxiated and/or having LBW. |
| Njim et al., 2017  Cameroon | To determine the prevalence of adolescent births and adverse outcomes as well as previous obstetric history. | Quantitative | Pregnant women:  <19y = 77  ≥ 19y = 809 | Previous obstetric history is not associated with adverse neonatal outcomes. |
| Bihoun et al., 2017  Burkina Faso | To assess the association between mother’s age and LBW and preterm birth. | Quantitative;  Prospective clinical trials | Pregnant women:  15-17y = 44  8-19y = 161  20-45y = 618 | Teenager’s pregnancy is associated with adverse fetal outcome in the rural setting, mainly when teenagers experience anemia and fever. |
| Yussif et al., 2017  Ghana | To explore the long-term effects of adolescent pregnancies on subsequent pregnancies and births on socioeconomic status. | Quantitative;  Cross-sectional survey | Pregnant women:  <19y = 46  ≥ 19y = 97 | Adolescent pregnancies [first or subsequent] are risk factors for adverse outcomes. |
| **Table 2: Extracted data and study characteristics (continued)** | | | | |
| Abbas et al., 2017  Egypt | To highlight the grave complications among teenage mothers compared with adult mothers. | Quantitative;  Prospective case control study | Pregnant women:  13 -19y = 606  20 – 29y = 2950 | Teenage mothers and their infants were more likely to experience complications than adults. |
| Laari et al., 2016  Ghana | To explore the factors that influence perinatal outcomes in adolescent pregnancies. | Quantitative;  Cross-sectional study | Pregnant women:  15-17y = 59  18-19y = 141 | The study indicates that mothers who are engaged in harmful traditional practices, had less than 37weeks gestation, used alcohol during pregnancy, never used family planning prior to their pregnancies and had increased odds of experiencing perinatal mortality. |
| Abdelsattar et al., 2016  Egypt | To evaluate teenage pregnancy outcomes | Quantitative;  Descriptive study | Pregnant women:  <19y = 694  >18y = 2814 | Teenage mothers are at higher risk of adverse maternal and neonatal outcomes compared to adult mothers and are more likely to be housewives and reside in rural areas. |
| Njim et al., 2016  Cameroon | To determine the prevalence and adverse outcomes of adolescent births. | Quantitative | Pregnant women:  ≤ 19y = 491  >19y = 4941 | Whether adolescents are married or not, their neonates are exposed to higher morbidity. This calls for adolescent-friendly policies to reduce adolescent pregnancies. |
| Mombo-Ngoma et al., 2016  Benin, Garbon, Mozambique & Tanzania | To assess whether young adolescent girls constitute a group at increased risk for adverse birth outcomes among pregnant women in SSA. | Quantitative;  Prospective clinical trial. | Pregnant women:  14 – 16y = 248  17 – 19y = 727  20 – 30y 2400  31+y = 725 | Young maternal age increases the risk for adverse pregnancy outcomes and it is a stronger predictor for low birth weight and preterm birth than other established risk factors in sub-Saharan Africa. |
| de Wet, 2016  South Africa | To examine levels and causes of adolescent maternal mortality in SA. | Quantitative | Pregnant women;  <20y = 1164 | Policies and programmes should prioritize these pregnancy-related causes of death in order to further reduce such deaths among adolescents. |
| Schipulle, 2015  Garbon | To assess the prevalence of adolescent pregnancies in semi-urban and rural settings and also analyze adolescence among other parameters as a potential risk factor for adverse pregnancy outcome focusing on LBW. | Quantitative;  Retrospective cohort study | Pregnant women:  <16y = 105  16-19y = 348  >19y = 1457 | Low frequency of antenatal care visits could be identified as independent risk factor for LBW and preterm birth. |
| Egbe et al., 2015  Cameroon | To determine the prevalence of teenage births and compare adolescent and adults on birth outcomes. | Quantitative;  Retrospect study. | Pregnant women:  14 – 19y = 148  20 – 29y = 360 | Adolescent pregnancies are more likely to lead to adverse fetal and maternal outcomes than adult pregnancies. |
| Ibrahim et al., 2015  Ghana | To compare mothers on LBW. | Quantitative | Pregnant women:  <18y = 12  18 – 24y = 428  25 – 34y = 802  >34 = 191 | Risk of adverse pregnancy outcome decreases with age. |
| Ngowa et al., 2015  Cameroon | To determine obstetrical and perinatal outcomes of nulliparous adolescent pregnancies. | Quantitative;  Retrospective cohort study | Pregnant women:  < 20y = 285  20 – 25y = 1394 | Adolescent pregnancy is associated with an increased risk of preeclampsia/ eclampsia, preterm birth and low birth weight. |
| Edessy et al., 2015  Egypt | To determine whether teenage pregnancy is associated with increased rates of adverse pregnancy outcomes. | Quantitative; Descriptive study | Pregnant women:  ≤ 19y = 1248  >19y = 245 | Teenage pregnancies are at higher risk of adverse pregnancy outcomes and they are likely to occur in illiterate housewives and from rural residence. |
| Fouelifack et al., 2014  Cameroon | To study the outcomes of adolescent pregnancies to inform public health actions. | Quantitative;  Cross-sectional study. | Pregnant women:  10 – 19y = 560  >19y = 5437 | Teenage pregnancies are associated with more referrals and poorer maternal and fetal outcomes compared to pregnancies in adults. Mothers in early adolescence [10–14 years] do not differ significantly in pregnancy out-comes with those in middle [15–17 years] or late adolescence [18–19 years]. |
| **Table 2: Extracted data and study characteristics (continued)** | | | | |
| Hougue et al., 2014  South Africa | To investigate adverse obstetric outcome in teenage pregnancy. | Quantitative; Retrospective comparative study. | Pregnant women:  <18y = 239  >17y = 2086 | This study highlighted that teenage mothers show similar obstetric outcome when compared with their adult counterparts |
| Ijarotimi et al., 2014  Nigeria | To examine the prevalence and outcome of teenage pregnancy. | Quantitative;  Retrospective study. | Pregnant women:  ≤ 15y = 10  16 – 19y = 245  >19y = 5995 | Optimal care should be given to teenage mothers not only to improve pregnancy outcome but also to enhance their social, educational and emotional adjustment. |
| Muganyizi et al., 2013  Tanzania | To examine risk factors and compare pregnancy outcomes in extreme reproductive ages. | Quantitative;  Retrospective study. | Pregnant women:  12-17y = 1680  35 – 50y = 7961 | There is rise in birth of mothers aged >35 years. Births at 35 years or above were on increase and were associated with the worst pregnancy outcomes in MNH. |
| Ganchimeg et al., 2013  Algeria, Angola, Kenya, Niger, Nigeria, and Uganda | To investigate the risk of adverse pregnancy outcomes and SC among adolescents in LMIC. | Quantitative: Cross-sectional study | Pregnant women:  <16y = 551  16 – 19y = 8998  20 – 24y = 10242  ≥ 25y = 7414 | There is a need for appropriate multidisciplinary interventions to prevent early pregnancies and to provide ANC and obstetric care for adolescent mothers in order to minimize their socio-economic deprivation and risk of adverse birth outcomes. |
| Ezegwui et al., 2012  Nigeria | To determine the current incidence of all teenage pregnancies and their obstetric outcomes. | Quantitative;  Retrospective study. | Pregnant women:  11-19y = 74  20 -34y = 105 | Teenage pregnancies are at higher risk than older counterparts. Female socio-economic development and proper use of contraceptive care will help reduce teenage pregnancy rate, while perinatal care will help to minimize its associated hazards. |
| Iklaki et al., 2012.  Nigeria | To determine the influence of ANC on perinatal outcome in teenage pregnancies. | Quantitative | Pregnant women:  ≤ 19y = 644 | Unbooked teenage pregnancies were significantly associated with increased operative intervention and poor perinatal outcome. |
| Obare et al., 2012  Kenya | To examine the factors associated with experiencing unintended pregnancies and poor birth outcomes and post-partum contraceptive use among HIV-positive female adolescents. | Quantitative;  Cross-sectional study. | Pregnant women:  <20y = 797 | This underscores the need for HIV and AIDS programs to provide appropriate sexual and reproductive health information and care to HIV-positive adolescents in order to reduce the risk of undesired reproductive health outcomes. |
| Ayuba et al., 2012  Nigeria | To evaluate risk factors associated with teenage pregnancy and compare obstetric and fetal outcome with older parturients. | Quantitative;  Retrospective study | Pregnant women:  14 -16y = 20  17 – 19y = 63  20 – 32y = 180 | Teenage pregnancy in the Niger Delta is concentrated among women with less formal education, who are unemployed, unmarried and with inadequate antenatal care and obstetric risks for poor pregnancy outcome. |
| Rasheed et al., 2011  Egypt | To determine the reasons for adolescent pregnancies and evaluate maternal, fetal and neonatal outcomes. | Quantitative. | Pregnant women:  < 20y = 2153  20 -30y = 3162 | Adolescent pregnancy increases the risk of ectopic pregnancy, pre-eclampsia, eclampsia, premature rupture of membranes, preterm birth and cesarean among mothers up to 16 years of age. After 16 years of age, pregnancy is not associated with increased risk of obstetric or neonatal complications. |
| Tebeu et al., 2011.  Cameroon | To test the hypothesis that births by teenagers were associated with increased risk. | Quantitative. | Pregnant women:  12 – 16y = 65  20 – 29y = 218 | Preterm birth remains significantly high even after adjustments of confounders. Findings underscore the importance of public health programs in preventing teenage pregnancy. |
| Adeyinka et al., 2010.  Nigeria | To evaluate risk factors of adolescent pregnancy and compare pregnancy complications of teens and adults. | Quantitative;  Retrospective study | Pregnancy women:  < 18y = 45  20 -35y = 90 | Psychological, nutritional and social work should be an integral part of obstetrical care in adolescent pregnancy, especially in low resource countries like Nigeria. |
| **Table 2: Extracted data and study characteristics (Continued)** | | | | |
| Kurth et al., 2010  Garbon | To investigate the influence of maternal adolescence on pregnancy outcomes. | Quantitative;  Cross-sectional study | Pregnant women:  ≤ 16y = 86  >16y = 689 | The study demonstrates the importance of adolescent age as risk factor for adverse pregnancy outcome. Antenatal care programs specifically tailored for the needs of adolescents may be necessary to improve the frequency of antenatal care visits and pregnancy outcomes in this risk group in Central Africa. |
| Omole_Ohonsi et al., 2010 | To review the obstetric outcome of teenage primigravid women. | Quantitative;  Retrospective case control. | Pregnant women:  < 20y = 500  20 -34y =500 | The results of this study show that teenage mothers who receive good family and community support, timely quality antenatal care and birth in hospitals, should expect similar obstetric outcome to that of their older peers. |
| Zeck et al., 2010  Tanzania | To compare adolescent pregnancy outcomes in Tanzania and Austria | Quantitative | Pregnant women:  12 -17y = 209  22 – 27y = 1341 | In contrast to the results in Tanzania, data from Austria show that the obstetric outcome in adolescent pregnancies can be favorable. However, socio-economic considerations have to be taken into account. Education and health knowledge seem critical for young females from low resource settings like Tanzania. |
| Hogue et al, 2010  South Africa | To estimate and compare the incidence of adverse obstetric and perinatal outcomes of teenage women with older women. | Quantitative;  Retrospective cohort study. | Pregnant women:  13-18y = 1236  19-46y = 6600 | Although there was a higher rate of teenage pregnancy, it did not appear that it was associated with extra perinatal negative outcome such as preterm birth, low birth-weight and stillbirth. There was also no difference in the extent of prenatal, birth and post-natal care. |
